# Intra-dialytic hypotension following the transition from continuous to intermittent renal replacement therapy

**DOI:** 10.1186/s13613-021-00885-7

**Published:** 2021-06-19

**Authors:** William Beaubien-Souligny, Yifan Yang, Karen E. A. Burns, Jan O. Friedrich, Alejandro Meraz-Muñoz, Edward G. Clark, Neill K. Adhikari, Sean M. Bagshaw, Ron Wald

**Affiliations:** 1 Division of Nephrology, Centre Hospitalier de L’Université de Montréal, Université de Montréal, 1000, rue St-Denis, Montreal, QC H2X 0C1 Canada; 2 Department of Medicine, University of Toronto, Toronto, ON Canada; 3 Interdepartmental Division of Critical Care Medicine, University of Toronto, Toronto, ON Canada; 4 Department of Health Research Methods, Evidence, and Impact, McMaster University, Hamilton, ON Canada; 5 Keenan Research Centre for Biomedical Science, Li Ka Shing Knowledge Institute, St. Michaels Hospital, Toronto, ON Canada; 6 Departments of Critical Care and Medicine, St. Michaels Hospital, University of Toronto, Toronto, ON Canada; 7 Division of Nephrology, St. Michaels Hospital and University of Toronto, Toronto, Canada; 8 Department of Medicine and Kidney Research Centre, Ottawa Hospital Research Institute, University of Ottawa, Ottawa, Canada; 9 Department of Critical Care Medicine, Interdepartmental Division of Critical Care, Sunnybrook Health Sciences Centre, University of Toronto, Toronto, Canada; 10 Department of Critical Care Medicine, Faculty of Medicine and Dentistry, School of Public Health, University of Alberta, Edmonton, Canada

**Keywords:** Renal replacement therapy, Acute kidney injury, Dialysis, Blood pressure, Hypotension, Hemodynamic instability

## Abstract

**Background:**

Transition from continuous renal replacement therapy (CRRT) to intermittent renal replacement therapy (IRRT) can be associated with intra-dialytic hypotension (IDH) although data to inform the definition of IDH, its incidence and clinical implications, are lacking. We aimed to describe the incidence and factors associated with IDH during the first IRRT session following transition from CRRT and its association with hospital mortality. This was a retrospective single-center cohort study in patients with acute kidney injury for whom at least one CRRT-to-IRRT transition occurred while in intensive care. We assessed associations between multiple candidate definitions of IDH and hospital mortality. We then evaluated the factors associated with IDH.

**Results:**

We evaluated 231 CRRT-to-IRRT transitions in 213 critically ill patients with AKI. Hospital mortality was 43.7% (*n* = 93). We defined IDH during the first IRRT session as 1) discontinuation of IRRT for hemodynamic instability; 2) any initiation or increase in vasopressor/inotropic agents or 3) a nadir systolic blood pressure of < 90 mmHg. IDH during the first IRRT session occurred in 50.2% of CRRT-to-IRRT transitions and was independently associated with hospital mortality (adjusted odds ratio [OR]: 2.71; CI 1.51–4.84, *p* < 0.001). Clinical variables at the time of CRRT discontinuation associated with IDH included vasopressor use, higher cumulative fluid balance, and lower urine output.

**Conclusions:**

IDH events during CRRT-to-IRRT transition occurred in nearly half of patients and were independently associated with hospital mortality. We identified several characteristics that anticipate the development of IDH following the initiation of IRRT.

**Supplementary Information:**

The online version contains supplementary material available at 10.1186/s13613-021-00885-7.

## Background

Renal replacement therapy (RRT) in the setting of acute kidney injury (AKI) in critically ill patients can be performed using continuous renal replacement therapy (CRRT) or intermittent renal replacement therapy (IRRT), which includes traditional intermittent hemodialysis and slow low efficiency dialysis (SLED). Although clinical trials comparing CRRT to IRRT have not demonstrated a definitive advantage on survival or recovery of kidney function [1–6], CRRT is recommended as the initial modality in critically ill patients with significant hemodynamic compromise [7]. In these patients, hemodynamic stability is theoretically enhanced by slow ultrafiltration rates and solute removal as compared to intermittent modalities where fluid removal and solute clearance occur at faster rates over shorter timeframes [8]. A recent international survey reported that two-thirds of practitioners reported using CRRT as the first modality in the ICU when fluid removal is indicated [9].

Patients who are perceived to have achieved hemodynamic stability and who still require RRT will frequently be transitioned from CRRT to intermittent modalities. However, a paucity of data exists about adverse events that may occur during these transitions. The Kidney Disease: Improving Global Outcomes (KDIGO) clinical practice guidelines for AKI recommend that this transition should be performed “once hemodynamic stability is achieved” [7], while others suggested urine output and correction of fluid overload should also be taken into consideration [10]. However, transitions to IRRT may be a context where intra-dialytic hypotension (IDH) and other adverse events are more likely to occur. In maintenance hemodialysis patients, IDH contributes to serious adverse events during sessions including myocardial stunning[11], cardiac arrhythmias [12], loss of residual kidney function [13], cerebral ischemia[14], intestinal ischemia [15], seizures [16] and cardiac arrest [17]. Although the dangers of IDH have been well-described in the maintenance dialysis population, there is limited information on the clinical implications of IDH in the setting of AKI [18].

The uncertainties surrounding RRT modality transitions in critically ill patients with AKI have been highlighted by the Acute Disease Quality Initiative (ADQI) [19]. In the present study, we sought to define IDH using relevant parameters and then describe its incidence, associated factors, and implications for patient outcomes after the transition to IRRT.

## Methods

### Patient selection

We conducted a single-center retrospective cohort study at St. Michael’s Hospital, a tertiary care academic hospital in Toronto, Canada. We queried the hospital’s AKI Registry [20], which contains demographic, clinical, physiological, and biochemical variables on all patients (*n* = 1213) who received acute RRT in one of four intensive care units (ICU) between April 1, 2007 and January 26, 2019. We identified patients for whom at least one modality transition from CRRT to IRRT took place. To be included, patients needed to have complete medical records, including ICU monitoring flowsheets, daily progress notes, and the IRRT prescription and session summary. We excluded transitions if the first IRRT session started outside of the ICU and for which the time gap between CRRT discontinuation and IRRT initiation was more than 7 days. If patients underwent multiple transitions within their ICU stay, data from all available transitions were included. The St. Michael's Hospital Research Ethics Board approved this study, which was performed in accordance with the 1964 Declaration of Helsinki and its subsequent amendments.

### Data collection

Baseline information included patient age at hospital admission, admission weight, and primary diagnostic category. We quantified the burden of comorbidities using the Charlson Comorbidity Index [21]. Information related to the receipt of RRT was collected including the time from hospital admission to the initiation of RRT, the number of CRRT-to-IRRT transitions in the ICU, and the time on CRRT before the transition to IRRT. Patient outcomes included hospital mortality, hospital discharge with RRT, as well as hospital and ICU length of stay.

For each CRRT-to-IRRT transition, we collected detailed clinical information at two time points: before the discontinuation of CRRT and immediately before the initiation of IRRT. Pharmacologic support including vasopressor and inotropic medications was quantified using the vasoactive-inotropic score (VIS) [22]. The severity of acute illness was summarized using the modified Sequential Organ Failure Assessment (SOFA) score as presented in Appendix 2 of the Additional file 1 [23]. Intermittent hemodialysis (IHD) and slow low efficiency dialysis (SLED) were defined as an IRRT session of fewer than 6 h and equal or more than 6 h, respectively. A complete list of collected variables is available in Additional file 1: Appendix 1.

### Data analysis

#### Association between IDH and hospital mortality

As there is no consensus definition for IDH in the setting of AKI [8, 24], we evaluated multiple pre-specified candidate definitions (Table 1). Core criteria for each definition included premature discontinuation of the first post-CRRT IRRT session related to hemodynamic instability and escalation of pharmacologic support during the IRRT session [24]. Escalation was defined by initiation of a new vasopressor/inotrope medication, or by a significant increase in the vasoactive-inotropic score (VIS) defined as either a ≥ 50% increase (Definition 1) or any increase (Definition 2). Other candidate definitions were created by integrating systolic (Definition 1A, 2A), or systolic and diastolic blood pressure (Definition 1B,2B) thresholds during IRRT.

**Table 1:** Candidate definitions of intra-dialytic hypotension during the first intermittent renal replacement therapy (IRRT) session performed in the intensive care unit after discontinuation of continuous renal replacement therapy

	Criteria (at least one)				Incidence within studied sample
	IRRT interruption	Pharmacologic support	Systolic blood pressure	Diastolic blood pressure	
Definition 1	Discontinuation of IRRT for instability	Initiation of new agent or ≥ 50% increase in VIS			28.6%
Definition 1A	Discontinuation of IRRT for instability	Initiation of new agent or ≥ 50% increase in VIS	Nadir of < 90 mmHg or, if starting sBP is < 90, a decrease of ≥ 10 mmHg		43.7%
Definition 1B	Discontinuation of IRRT for instability	Initiation of new agent or ≥ 50% increase in VIS	Nadir of < 90 mmHg or, if starting sBP is < 90, a decrease of ≥ 10 mmHg	Nadir of < 40 mmHg or, if starting dBP is < 40 mmHg. a decrease of ≥ 5 mmHg	46.8%
Definition 2	Discontinuation of IRRT for instability	Initiation of new agent or any increase in VIS			38.1%
Definition 2A	Discontinuation of IRRT for instability	Initiation of new agent or any increase in VIS	Nadir of < 90 mmHg or, if starting sBP is < 90, a decrease of ≥ 10 mmHg		50.2%
Definition 2B	Discontinuation of IRRT for instability	Initiation of new agent or any increase in VIS	Nadir of < 90 mmHg or, if starting sBP is < 90, a decrease of ≥ 10 mmHg	Nadir of < 40 mmHg or, if starting dBP is < 40 mmHg. a decrease of ≥ 5 mmHg	52.8%

*dBP* diastolic arterial blood pressure, *sBP* systolic arterial blood pressure, *VIS* vasoactive-inotropic score

The association between candidate definitions of IDH and hospital mortality was assessed using generalized estimating equation (GEE) models with logistic link function using an M-estimator for the covariance matrix and an independent structure for the working correlation matrix. This type of analysis accounts for the repeated measures design because multiple CRRT-to-IRRT transitions occurred in some patients. Results are presented as odds ratios (OR) with 95% confidence intervals (CI). For each candidate variable, a first model including the candidate definitions as a binary variable was constructed. A second multivariable model was constructed by adding the VIS score at the start of the IRRT session, mechanical ventilation during the IRRT session and Charlson comorbidity score to adjust for the patient baseline status and severity of illness before IRRT initiation. Interactions between variables were tested with a significance level of *p* < 0.05. In a sensitivity analysis, we added IRRT modality (IHD vs SLED) as an adjustment variable for the association between IDH and in-hospital mortality using the same aforementioned methodology. We also tested for interaction between IDH and the IRRT modality.

Because the magnitude of the association between candidate definitions for IDH and mortality had overlapping confidence intervals, we selected the most appropriate IDH definition by selecting the model with the best goodness of fit. We selected the definition of IDH resulting in the lowest quasi-likelihood information criterion (QIC), which is used to compare model fit in GEE models [25]. Given the potential limitations of this approach [26], we also performed a sensitivity analysis using only data from the first transition in the studied patients to construct logistic regression models and compared several indicators of goodness of fit including Nagelkerke [27] and Cox/Snell pseudo-R^2^ [28]. In case of equality, we favored the simplest definition.

#### Association between clinical variables and IDH

We assessed the association between clinical variables and IDH using GEE at two separate time points: the time of CRRT discontinuation and the time of IRRT initiation. The association for each clinical variable was first assessed in univariate analysis and two multivariable models were then constructed. The first one contained variables available at CRRT discontinuation while the second contained variables available at the time of IRRT initiation. For continuous variables, the Box-Tidwell test was used to verify the assumption of linearity [29]. The ability of the multivariable models to predict IDH in the derivation cohort was assessed using the area under the receiver-operating characteristic curve (C-statistic) with 95% confidence intervals. Additionally, the performance of the models was assessed in patients who received conventional IHD as the first IRRT modality. During analysis, the VIS before the start of the IRRT session did not satisfy the Box-Tidwell criteria for the linearity assumption and therefore the use of vasopressor medication was included as a binary variable (yes/no). In an exploratory analysis, restricted cubic splines regression with knots placed at the quintiles of VIS distribution was used to model the relationship between the predicted risk of IDH and the VIS before the start of the IRRT session. We performed the same analysis in the CRRT-to-IDH and the CRRT-to-SLED subgroups, as well as for the association between prescribed fluid removal and IDH.

In a supplementary analysis, we assessed for statistical interactions with the time period in which the transition occurred (each year since 2007) and all clinical variables included in the model. An interaction was considered significant if the *p*-value was < 0.05. We also verified if an association was present between the time period and IDH, and between the change in SOFA score during the transition period and IDH.

We present descriptive data as numbers (%) for dichotomous variables and as mean ± standard deviation (SD) or, alternatively, median and interquartile range (IQR) for continuous variables, where appropriate. Analyses were conducted in SPSS 25 (IBM, Armonk) and R (R core team, Vienna). Results are reported according to the Strengthening the Reporting of Observational Studies in Epidemiology (STROBE) statement [30].

## Results

### Characteristics of the cohort

We identified 213 eligible patients with 231 transitions from CRRT to IRRT (Additional file 1: Figure S1). Patient characteristics are presented in Table 2. The majority of patients (198 patients, 93.0%) had one transition during their hospital stay. Hospital mortality was 93 (43.7%), and among survivors, 31 (14.6%) remained RRT-dependent at hospital discharge.

**Table 2:** Patient characteristics

Characteristics	*N* = 213
Female sex	64 (30.0%)
Category	
Medical	120 (56.3%)
Surgical	93 (43.7%)
Age (years)	62.7 (52.8; 72.2)
Admission weight (kg)	85 (71; 101)
Baseline estimated glomerular filtration rate	46 (25; 74)
Comorbidities	
Myocardial infarction	27 (12.7%)
Diabetes	65 (30.5%)
Congestive heart failure	34 (16.0%)
Peripheral artery disease	17 (8.0%)
Chronic lung disease	43 (20.2%)
Malignancy	30 (14.1%)
Moderate to severe liver disease	17 (8.0%)
Charlson score	2 (1; 4)
Time from hospital admission to RRT (days)	5 (2; 12)
Number of transitions attempts	
1	198 (93.0%)
2	12 (5.6%)
3	3 (1.4%)
Time on CRRT before transition attempt (days)	5 (3; 10)
Time gap between CRRT and IRRT (h)	24.5 (15.3; 41.2)
Outcomes	
Death in the ICU	82 (38.5%)
Death in hospital	93 (43.7%)
Discharged from hospital without RRT	89 (41.8%)
Discharged from hospital with RRT	31 (14.6%)
Length of stay in the ICU (days)	23 (14; 42)
Length of hospital stay (days)	38 (24; 65)

	At CRRT discontinuation	Before IRRT
Severity of illness (*n* = 231 transitions)		
Total SOFA score^b^	9 (SD:4)	10 (SD:3)
Vasopressor use	95 (41.1%)	102 (44.2%)
VIS	0 (IQR: 0; 7.5)	0 (IQR: 0; 8.0)
Mechanical ventilation	205 (88.7%)	201 (87.0%)
Cumulative fluid balance (liters)	7.4 (IQR: 1,5; 14.1)	7.6 (IQR: 2.3; 15.2)
Fluid accumulation (% of body weight)	8.3 (1.7; 16.7)	8.0 (2.7; 17.7)

Data are presented in mean (standard deviation (SD)) or median (interquartile range (IQR)), where appropriate

*CRRT* continuous renal replacement therapy, therapy, *IRRT* Intermittent renal replacement therapy, *VIS* vasoactive-inotropic score

^a^Number of times a transition from CRRT-to-IRRT occurred during intensive care unit (ICU) stay

^b^Detailed components of the sequential organ failure assessment (SOFA) score are presented in Tables S5 of the Additional file 1: Appendix

Patient characteristics at CRRT discontinuation and IRRT initiation are presented in Table 2. Most patients were mechanically ventilated (88.7% and 87.0%, respectively) and a substantial proportion was receiving vasopressor support (41.1% and 44.2%, respectively). The median period between CRRT discontinuation and IRRT initiation was 24.5 (IQR: 15.3; 41.2) hours. We present IRRT parameters, intra-session events, and events during the 72 h after the session in Additional file 1: Table S1. SLED was used as the initial IRRT modality in 87 (37.7%) transitions. IRRT was prematurely interrupted for clinical events in 6 (2.6%) sessions including cardiac arrest (*n* = 1), urgent endotracheal intubation (*n* =  1) and severe hemodynamic instability (*n* = 4).

### Selecting a definition for intra-dialytic hypotension

The incidence of IDH ranged from 29 to 53% depending on the candidate definition utilized (Table 1). Associations between candidate definitions of IDH and hospital mortality are presented in Table 3. All candidate definitions were significantly associated with hospital mortality in univariable and adjusted analyses. However, definitions including “any increase in VIS” as a criterion (definition 2, 2A, 2B) were more strongly associated with hospital mortality than definitions considering only “a relative increase of 50% or more in VIS” (definition 1, 1A, 1B) and produced models with better goodness of fit (Additional file 1: Table S2). Adding the sBP criteria to definition 2 (corresponding to definition 2A) led to improvement in the ‘goodness of fit’ of the model (Additional file 1: Figure S2). However, adding the dBP criteria (corresponding to definition 2B) did not improve the ‘goodness of fit’ of the model while adding to the operational complexity of the definition. Consequently, we established definition 2A (composite of RRT discontinuation for hemodynamic instability, vasopressor escalation or SBP decline to < 90 mmHg or ≥ 10 mmHg decline if pre-RRT SBP < 90), which occurred in 50.2% of transitions, as the definition of IDH for subsequent analyses. IDH was not significantly associated with RRT at hospital discharge (OR: 1.17 (CI 0.51; 2.68) *p* = 0.706) (Additional file 1: Table S3). Clinical variables in relationship with the occurrence of IDH are presented in Additional file 1: Table S4.

**Table 3:** Association between candidate definitions of intra-dialytic hypotension and hospital mortality

	Crude OR (95%CI) *p*-value^a^	Adjusted OR (95%CI) *p*-value^b^
Criteria 1	2.09 (1.17; 3.73) 0.013	1.89 (1.02; 3.52) 0.043
Criteria 1A	2.45 (1.44; 4.16) 0.001	2.39 (1.35; 4.25) 0.003
Criteria 1B	2.39 (1.40; 4.08) 0.001	2.28 (1.29; 4.03) 0.005
Criteria 2	3.30 (1.90; 5.73) < 0.001	2.46 (1.36; 4.48) 0.003
Criteria 2A	3.35 (1.92; 5.83) < 0.001	2.71 (1.51; 4.84) 0.001
Criteria 2B	3.42 (1.96; 5.95) < 0.001	2.73 (1.52; 4.89) 0.001

Associations were assessed using generalized estimating equations with a binary logistic link function and using an M-estimator with an independent correlation matrix

*CI* 95% confidence intervals

*OR* odds ratio

^a^Model including the candidate definitions as a binary variable

^b^Model with adjustment for vasoactive-inotropic score at the start of IRRT session, mechanical ventilation during IRRT session and Charlson comorbidity score

### Factors associated with IDH at the time of CRRT discontinuation

**Fig. 1:**
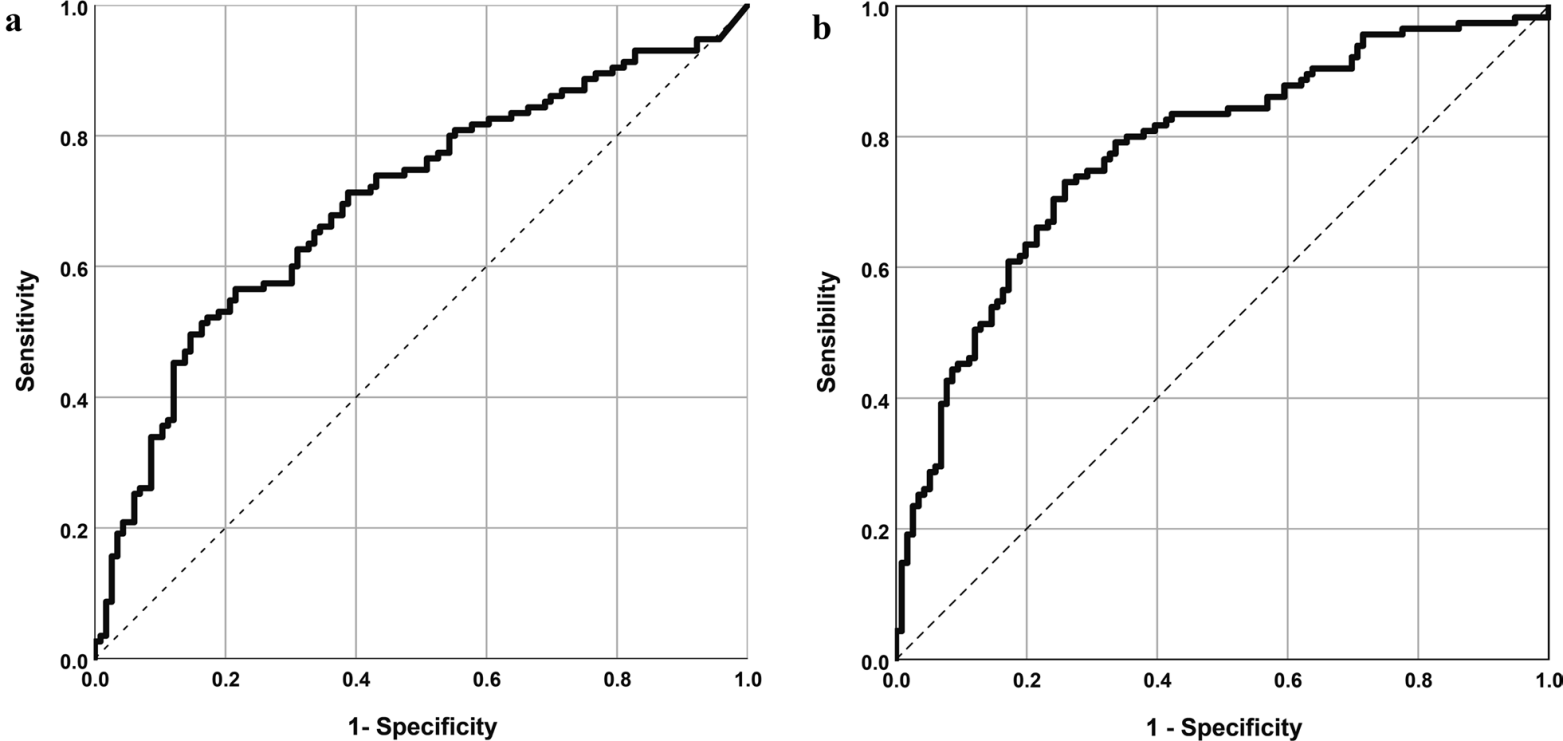
Receiver operating characteristic curve illustrating the ability to predict intra-dialytic hypotension during the first intermittent renal replacement therapy session after discontinuation of continuous renal replacement therapy (CRRT). **A** Multivariable model combining variables available at CRRT discontinuation (AUC: 0.70 CI: 0.63; 0.77 *p* < 0.001). **B** Multivariable model combining variables available immediately before IRRT initiation (AUC: 0.78 CI: 0.72; 0.84 *p* < 0.001**)**

Several clinical variables, recorded at the time of CRRT discontinuation, were significantly associated with IDH during the first IRRT session including cumulative fluid balance (OR: 1.03 (CI: 1.01; 1.06) *p* = 0.009 per L), 24-h urine output (OR: 0.91 (CI 0.83; 0.999) *p* = 0.047 per 100 mL of urine) and receipt of any vasopressor (OR: 3.16 (CI 1.80; 5.54) *p* < 0.001) (Table 4). The associations remained significant after multivariable adjustment. The resulting multivariable model had a fair ability to predict IDH within the cohort (AUC: 0.70 CI 0.63; 0.77 *p < *0.001) (Fig. 1A).

**Table 4:** Variables associated with intra-dialytic hypotension (using Definition 2A)

Variable	Univariable OR (95%CI) *p*-value	Multivariable Adj OR (95%CI) *p*-value
Model 1: before CRRT discontinuation		
Mechanical ventilation	1.87 (0.79; 4.44) 0.155	1.35 (0.47; 3.89) 0.579
Cumulative fluid balance (per L)	1.03 (1.01; 1.06) 0.009	1.04 (1.01; 1.06) 0.013
24-h urine output (per 100 mL)	0.91 (0.83; 0.999) 0.047	0.90 (0.82; 0.98) 0.017
Time on CRRT (per day)	1.04 (0.99; 1.08) 0.093	1.04 (0.99; 1.10) 0.095
Vasopressor use	3.16 (1.80; 5.54) < 0.001	3.29 (1.84; 5.89) < 0.001
Number of past transitions attempts	1.96 (0.99; 3.88) 0.052	1.08 (0.46–2.52) 0.856
Model 2: before IRRT initiation		
Heart rate (per 10 beats/min)	1.06 (0.92; 1.21) 0.432	1.07 (0.90; 1.27) 0.428
sBP (per 10 mmHg)	0.75 (0.65; 0.86) < 0.001	0.85 (0.73; 0.999) 0.05
dBP (per 10 mmHg)	0.65 (0.51; 0.82) < 0.001	0.79 (0.59; 1.05) 0.107
Mechanical ventilation	2.93 (1.24; 6.89) 0.014	2.12 (0.85; 5.29) 0.109
Vasopressor use	3.95 (2.26; 6.91) < 0.001	2.22 (1.11; 4.43) 0.024
Prescribed relative fluid removal (% of BW)	1.76 (0.25; 12.35) 0.571	1.26 (1.01; 1.59) 0.043
Time gap between CRRT and IRRT (days)	0.74 (0.57; 0.97) 0.029	0.99 (0.98; 1.00) 0.162
Prescribed treatment time (hours)	1.51 (CI: 1.29; 1.76) < 0.001	1.33 (1.12; 1.59) 0.001

Associations were assessed using generalized estimating equations with a binary logistic link function and using an M-estimator with an independent correlation matrix

*Adj* adjusted, *BW* body weight, *95%CI* 95% confidence interval, *CRRT* continuous renal replacement therapy, *dBP* diastolic arterial blood pressure, *IRRT* intermittent renal replacement therapy, *OR* odds ratio, *sBP* systolic arterial blood pressure

### Factors associated with IDH at the time of IRRT initiation

At the time of IRRT initiation, the following variables were significantly associated with subsequent IDH: sBP before the start of IRRT (OR: 0.75 (CI 0.65; 0.86) *p* < 0.001 per 10 mmHg increase), dBP before the start of IRRT (OR: 0.65 (CI 0.51; 0.82) *p* < 0.001 per 10 mmHg increase), mechanical ventilation (OR: 2.93 (CI 1.24; 6.89) *p* = 0.014), vasopressor use (OR: 3.95 (CI 2.26; 6.91) *p* < 0.001), the time elapsed between CRRT discontinuation and IRRT initiation (OR: 0.74 (CI 0.57; 0.97) *p* = 0.029 per day) and prescribed treatment time (OR: 1.51 (CI 1.29; 1.76) *p* < 0.001 per h of treatment) (Table 4).

In multivariable analysis, sBP, vasopressor use, and prescribed treatment time remained independently associated with IDH. While prescribed relative fluid removal by itself was not associated with IDH in univariable analysis, a significant association was observed after adjustment for other variables in the model (aOR: 1.26 (1.01; 1.59) *p* = 0.043 per % of BW). The resulting model had a fair ability to predict IDH within the cohort (AUC: 0.78 CI: 0.72–0.84 *p* < 0.001) (Fig. 1B).

When considering the association between the VIS before initiation of IRRT and the risk of IDH, restricted spline regression analysis suggested a non-linear relationship with an important increase in the probability of IDH associated with the initiation of pharmacologic support (VIS 0 to 5) while further elevation in VIS was not associated with an increase in the probability of IDH (Additional file 1: Figure S2).

Of interest, elements related to IRRT prescription, including dialysate temperature and sodium concentration, were not associated with IDH (Additional file 1: Table S4).

### Analysis for CRRT-to-IHD subgroup and other sensitivity analyses

When considering only CRRT-to-IHD transitions (*n* = 144), associations between clinical variables and IHD were generally consistent with the whole cohort (Additional file 1: Table S6). The predictions models performed similarly in this subgroup (Model 1: AUC: 0.65 (CI 0.56; 0.74)* p* = 0.003 and Model 2: AUC: 0.73 (CI: 0.64; 0.82) *p* < 0.001).

Disease severity was generally higher during CRRT-to-SLED transitions compared to CRRT-to-IDH transitions (Additional file 1: Table S7). CRRT-to-SLED transitions were associated with a higher risk of in-hospital mortality compared to CRRT-to-IDH transition (OR: 3.13; (1.72; 5.68) *p* < 0.001). However, the association between IDH and in-hospital mortality remained after adding the receipt of SLED as an adjustment variable (OR: 2.54 CI 1.41; 4.58 *p* = 0.002). There was no interaction between IDH and the receipt of SLED (*p* = 0.876).

When replacing the prescribed treatment duration with the receipt of SLED in IDH prediction Model 2, the associations remained consistent (Additional file 1: Table S8). The revised prediction model did not perform better than the original Model 2 (AUC: 0.766 (0.704; 0.827) *p* < 0.001).

There was no significant interaction between the time period (year) at which the transition occurred and each clinical variable included in the IDH prediction models. Furthermore, there was no association between the time period and IDH (OR: 1.03 CI: 0.96; 1.11 *p* = 0.391 per year since 2007).

Finally, there was no association between the change in SOFA score between the discontinuation of CRRT and the initiation of IRRT (OR: 1.04 CI: 0.93; 1.16 *p* = 0.481 per 1 point increase).

## Discussion

In critically ill patients with acute kidney injury, IDH occurring after the transition from CRRT to IRRT was common and independently associated with hospital mortality. We identified multiple clinical risk factors for IDH based on information available to the clinician at the time of CRRT discontinuation as well as immediately before the initiation of IRRT. These factors may assist clinicians in identifying patients at risk for hemodynamic instability during IRRT treatment.

Many considerations justify transitions from CRRT to IRRT. The use of CRRT leads to increased costs while its clinical benefit has not been demonstrated [31]. Furthermore, although physical therapy can be conducted while the patient received CRRT [32], transition to IHD also greatly simplify patient mobilization which represents a fundamental component of patient rehabilitation after critical illness [33]. However, even though RRT modality transitions are frequent events in the ICU, limited data exist regarding adverse events occurring during RRT modality transitions in critically ill patients. In a retrospective study, Jeon et al. studied the outcome of 1176 patients who attempted discontinuation of CRRT in the ICU [34]. In their cohort of patients who resumed RRT, 310 (26.4%) started IRRT and 349 (29.7%) re-started CRRT. However, the reasons for CRRT re-initiation, as well as adverse events that occurred during the transition from CRRT to IRRT were not described.

In the present study, we observed that IDH occurred in more than half of patients during the first IRRT session. IDH events negatively impact the quality of delivered RRT and may influence patient outcomes. Among critically ill patients receiving RRT, mean arterial pressure during RRT is associated with an increase in the risk of hospital mortality [3, 35] and a lower likelihood of kidney function recovery [3]. In the recently concluded STARRT-AKI trial, accelerated initiation of RRT conferred greater dependence at 90 days [36]. This may have been mediated by IDH which was also more common in that treatment arm. Beyond what occurs during critical illness, further episodes of IDH during the recovery period may also hamper kidney recovery [37, 38].

When considering IDH events, arterial blood pressure values alone do not provide a complete picture of hemodynamic status since pharmacologic support is often utilized in critically ill patients. At present, there is no consensus regarding the most appropriate definition of IDH in an ICU setting. We therefore tested multiple a priori candidate definitions and selected the most clinically relevant definition using a pre-specified analytic approach. The selected definition of IDH comprised a marker of clinical relevance (premature RRT discontinuation), vasopressor use and objective drops in sBP and thus may be better suited to patients who require RRT in the ICU environment [39].

Vasopressor use, both at the time of CRRT discontinuation and at IRRT initiation, was associated with IDH. Specifically, the receipt of any vasopressor support, irrespective of the specific dose, was strongly associated with IDH. We observed that a higher cumulative fluid balance at CRRT discontinuation is associated with IDH. Similarly, prescribed relative fluid removal was associated with IDH when treatment time was included as an adjustment variable. While a high net fluid removal rate has been associated with adverse events in maintenance hemodialysis patients [40], data is scarce related to critically ill patients in whom tolerance to fluid removal may vary widely between individuals. Beyond routinely available clinical information, predicting tolerance to fluid removal may require adjunct information including dynamic assessment of preload responsiveness at the bedside [41] and other sources of information. In a recent study, a combination of cardiovascular SOFA score, capillary refill time and serum lactate achieved moderate performance in predicting hemodynamic instability [42].

Our study has several strengths. First, this is the first report to specifically examine adverse events during CRRT-to-IRRT transitions in critically ill patients. Second, we used an institutional AKI database that precisely recorded when RRT modality transitions occurred. This approach ensured that we could identify all transition events within the study period, thereby reducing the risk of selection bias. Third, we collected detailed information regarding the hemodynamic status and vasopressor use as well as characteristics of IRRT. Finally, in the absence of prior consensus, we identified the most appropriate definition of IDH using a data-driven approach instead of using an arbitrary definition.

Our study also has limitations. First, this is a retrospective study in a single center which may limit generalizability. Because of the option of transitioning to SLED, which may be better tolerated than IHD, our findings may not apply to centers that do not offer SLED and where patients transition directly from CRRT to IHD. Furthermore, while CRRT remains the preferred modality in hemodynamically unstable patients [9], whether it is better tolerated than SLED remains unproven and this question was not explored in the present work since we lacked detailed hemodynamic data in the period leading to the discontinuation of CRRT. Most importantly, we did not compare hemodynamic parameters to a control group composed of patients that remained of CRRT. Consequently, we cannot determine if the decision to continue CRRT instead of transitioning to IRRT would have prevented IDH. Most importantly, although IDH is associated with adverse outcomes in the setting of acute and maintenance HD, we cannot assume a causal link with adverse outcomes. Additionally, IDH definition was based on the arterial blood pressure nadir during treatment which may have been transient. The duration and frequency of hypotensive episodes during hemodialysis may carry prognostic information but was not captured in the present work. Similarly, we did not include important information about the trajectory of critical illness leading up to the transition. Finally, the IDH prediction models in our study only performed moderately well within the development cohort. These models require further evaluation in other databases to confirm our findings. Furthermore, the small number of patients included in our study may have reduced our ability to observe other significant associations between potential predictors of IDH and relevant outcomes such as kidney recovery.

## Conclusions

Patients frequently experience IDH during the first IRRT session after transitioning from CRRT and the events are independently associated with an increased risk of hospital mortality. IDH episodes may be anticipated using clinical characteristics before CRRT discontinuation and before IRRT initiation. Future research is needed to further clarify the clinical implications of IDH in the context of modality transitions.

## Supplementary Information

**Additional file 1**. Supplementary material including Appendix 1, Appendix 2, Figures S1 and S2, Tables S1 to S8.

## Data Availability

The datasets used and/or analyzed during the current study are available from the corresponding author on reasonable request.

## Abbreviations

ADQI: Acute disease quality initiative
AKI: Acute kidney injury
CI: Confidence intervals
CRRT: Continuous renal replacement therapy
GEE: Generalized estimating equations
ICU: Intensive care unit
IHD: Intermittent hemodialysis
IDH: Intra-dialytic hypotension
IQR: Interquartile range
IRRT: Intermittent renal replacement therapy
KDIGO: Kidney Diseases: Improving Global Outcomes
sBP: Systolic blood pressure
dBP: Diastolic blood pressure
SLED: Slow low efficiency hemodialysis
STROBE: Strengthening the Reporting of Observational Studies in Epidemiology
QIC: Quasi-likelihood information criterion
OR: Odds ratio
VIS: Vasoactive-inotropic score
