# Supplementary material for: Intra-dialytic hypotension following the transition from continuous to intermittent renal replacement therapy
Source: Ann Intensive Care. 2021 Jun 19;11:96. doi: 10.1186/s13613-021-00885-7 (PMC8214642; doi:10.1186/s13613-021-00885-7)
Supplement: Supplementary file 1 — Additional file 1. Supplementary material including Appendix 1, Appendix 2, Figures S1 and S2, Tables S1 to S8. [file 13613_2021_885_MOESM1_ESM.docx]

**Supplementary material**

**Appendix 1: Data collected**

At both timepoints, information was collected about pharmacologic support (vasopressors and inotropes), mechanical ventilation and fluid balance including 24-hour urine output, 24-hour fluid balance and cumulative fluid balance since ICU admission. The interval between CRRT discontinuation to the initiation of IRRT was recorded. Heart rate, systolic arterial blood pressure (sBP) and diastolic arterial blood pressure (dBP) before the initiation of IRRT were collected. Prescribed dialysis parameters including planned fluid removal, planned treatment duration, dialysate sodium concentration and dialysate temperature were collected. Fluid removal was expressed in % of body weight (BW) at hospital admission.

During the first IRRT session, information was collected from the dialysis summary and the ICU nursing flowsheets. The nadir sBP and dBP were collected as well as whether vasoactive agents were initiated or increased during the IRRT session. The total fluid removal achieved during the session and the actual duration of the IRRT session were collected. Events including IRRT discontinuation for clinical events (severe hypotension, cardiac arrest, respiratory failure) or technical factors (clotting, vascular access problems) were collected. Adverse events potentially related to IDH including death, cardiac arrest, stroke, seizures, myocardial infarction and mesenteric ischemia occurring within 72 hours of the first IRRT session were collected.

The vasoactive-inotropic score (VIS) was defined as [VIS =dopamine dose + dobutamine dose + 100*epinephrine dose + 10*milrinone dose + 10000*vasopressin dose + 100*norepinephrine dose] where all doses are in µg/kg/min except vasopressin dose which is in units/kg/min [20].

**Appendix 2: SOFA score**

Sequential organ failure assessment (SOFA) score[36]

| Points | 0 | +1 | +2 | +3 | +4 |
| --- | --- | --- | --- | --- | --- |
| PaO2/FiO2 | ≥ 400 | 300-399 | 200- 299 | 100- 199 | <100 |
| Glasgow Coma Scale | 15 | 13-14 | 10-12 | 6-9 | <6 |
| Cardiovascular | MAP ≥ 70 | MAP <70 | dopamine ≤ 5 µg/kg/min or dobutamine (any dose) | dopamine > 5 µg/kg/min OR epinephrine ≤ 0.1 µg/kg/min OR norepinephrine ≤ 0.1 µg/kg/min | dopamine > 15 µg/kg/min OR epinephrine > 0.1 µg/kg/min OR norepinephrine > 0.1 µg/kg/min |
| Bilirubin (µmol/L) | <20 | 20-32 | 33-101 | 102-204 | >204 |
| Platelets (x10^3^/µL) | ≥ 150 | 149-100 | 99-50 | 49-20 | <20 |
| Creatinine (µmol/L) | <110 | 110-170 | 171-299 | 300-440 | >440 |

Legend: PaO_2_: Partial blood oxygen pressure, FiO_2_ : Inspired oxygen fraction, MAP: mean arterial pressure.

**Figure S1: Inclusion flowchart. Abbreviations: CRRT: Continuous renal replacement therapy, ICU: Intensive care unit, IRRT: Intermittent renal replacement therapy,**


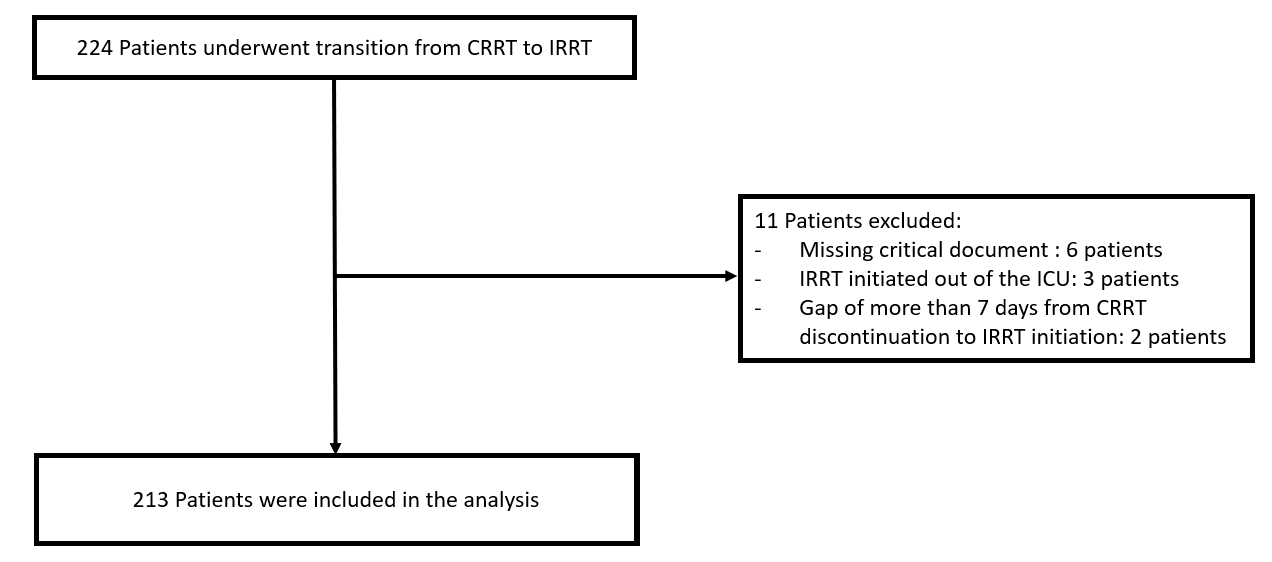


**Figure S2: Restricted cubic spline regression illustrating the relationship between the predicted probability of intra-dialytic hypotension (IDH) during the first intermittent renal replacement therapy (IRRT) session and the vasoactive-inotropic score (VIS) before IRRT initiation in all transitions (A), CRRT-to-IHD transitions (B) and CRRT-to-SLED transitions (C), as well as with the prescribed fluid removal in all transitions (D). Five knots were placed at quintiles of distribution and 95% confidence intervals are shown in grey. The results suggest a non-linear relationship with an important increase in the probability of IDH associated with the initiation of pharmacologic support (VIS 0 to 5) while further elevation in VIS was not associated with an increase in the probability of IDH.**


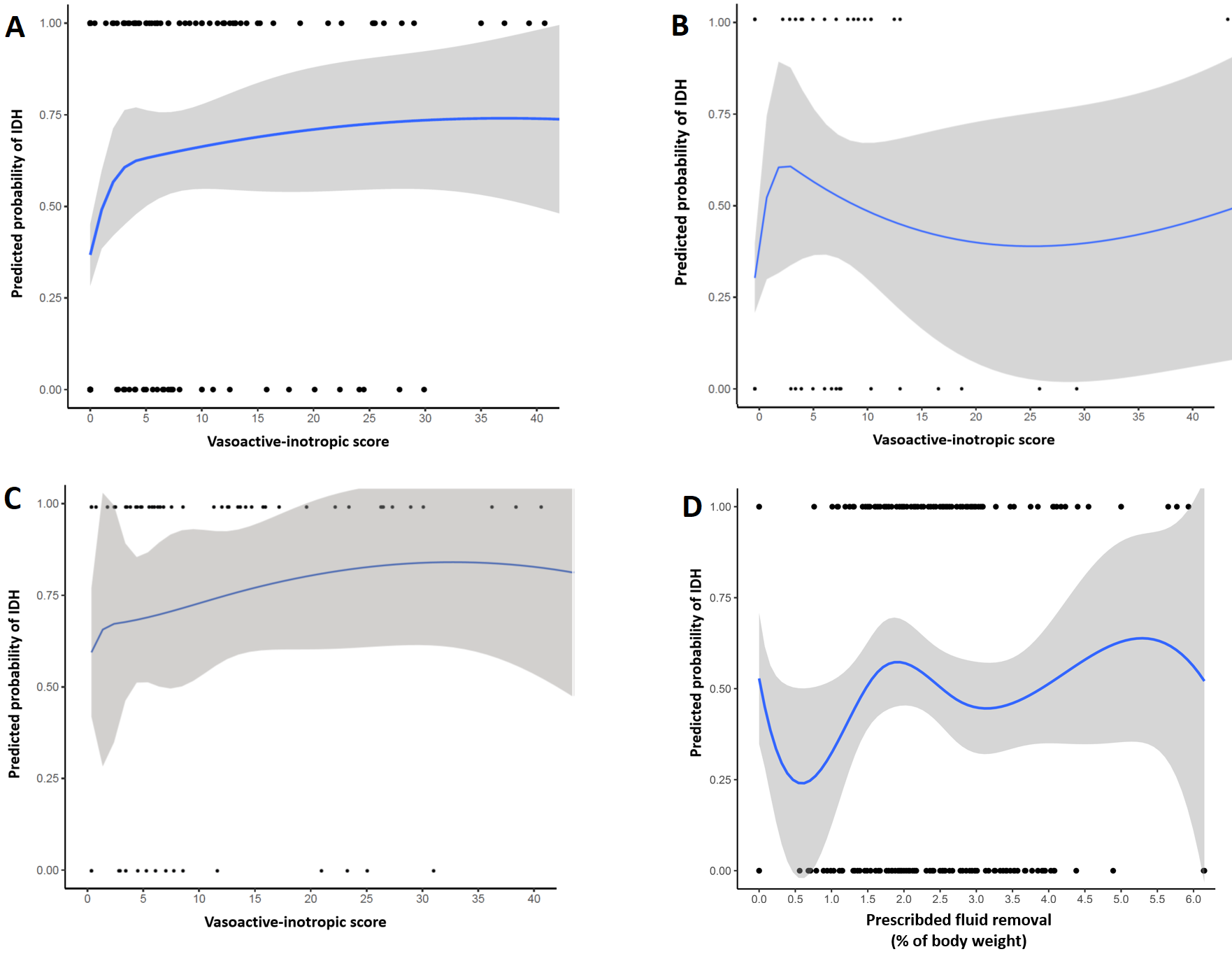


**Table S1: Characteristics and clinical events during and after the first intermittent renal replacement therapy session after discontinuation of continuous renal replacement therapy.**

| **Dialysis parameter** | |  |  |
| --- | --- | --- | --- |
|  | | **Prescribed** | **Achieved** |
| **Treatment time** | | 4.0 (4.0; 7.6) | 4.0 (3.8; 7.5) |
| **Relative fluid removal (%BW)** | | 2.14 (1.36; 2.99) | 2.00 (1.21; 3.09) |
| **SLED modality (treatment duration >6 hours)** | | 87 (37.7%) | |
| **Anticoagulation use** | | 64 (27.7%) | |
| **Blood flow rate** | **Prescribded** | 250 (200; 300) | |
|  | **Acheived** | 250 (200; 300) | |
| **Dialysate sodium** | **≤ 135 mmol/L**  **136-140 mmol/L**  **>140 mmol/L**  **Ramping** | 33 (14.3%)  168 (72.7%)  9 (3.9%)  22 (9.5%) | |
| **Dialysate temperature** | **<36.0**  **≥36.0**  **Blood temperature monitor** | 22 (9.5%)  195 (84.4%)  14 (6.1%) | |
| **Premature interruption** | **For technical issues**  **For clinical events during dialysis**   - **Severe hypotension** - **Urgent intubation** - **Cardiac arrest** | 15 (6.5%)  6 (2.6%)  4 (1.7%)  1 (0.4%)  1 (0.4%) | |
| **Adverse events ≤72 hours after first IRRT session** | **Death**  **Myocardial infarction**  **Seizure**  **Stroke**  **Bowel ischemia** | 8 (3.5%)  1 (0.4%)  0 (0%)  0 (0%)  1 (0.4%) | |
|  | **Re-initiation of CRRT** | 17 (7.4%) | |

Legend: BW: body weight at admission, SLED: slow low efficiency dialysis

**Table S2: Goodness-of-fit measures for models predicting in hospital mortality based on candidate definitions of intra-dialytic hypotension**

| Definition  of IDH | GEE model  (231 transitions) |  | Logistic regression model  (213 transitions) | |
| --- | --- | --- | --- | --- |
|  | **Quasi-likelihood under**  **the independence criterion (QIC)*** | **Odds ratio**  **(95%CI) p** | **Nagelkerke’s**  **pseudo-R^2^** | **Cox Snell’s**  **pseudo-R^2^** |
| 1 | 316 | 2.31 (1.25-4.26) p=0.008 | 0.05 | 0.03 |
| 1A | 311 | 3.09 (1.75-5.45) p<0.001 | 0.10 | 0.07 |
| 1B | 311 | 3.12 (1.78-5.49) p<0.001 | 0.10 | 0.07 |
| 2 | 303 | 3.66 (2.03-6.58) p<0.001 | 0.12 | 0.09 |
| 2A | 302 | 4.15 (2.34-7.39) p<0.001 | 0.15 | 0.11 |
| 2B | 302 | 4.25 (2.38-7.58) p<0.001 | 0.15 | 0.11 |

Legend: The logistic regression models only include data from the first transition attempt in each patient. *A lower QIC indicates better goodness-of-fit.

**Table S3: Association between adverse outcomes and intra-dialytic hypotension during the first intermittent renal replacement therapy (IRRT) after continuous renal replacement therapy (CRRT).**

| Outcomes | OR (CI) p-value |
| --- | --- |
| Failure of modality transition: death or re-initiation of CRRT within 72 hours | 1.78 (CI: 0.75; 4.26) p= 0.193 |
| CRRT re-initiation within 72 hours | 2.59 (CI: 0.88; 7.60) p= 0.084 |
| Death within 72 hours | 0.77 (CI: 0.19; 3.21) p= 0.724 |
| RRT at hospital discharge | 1.17 (CI: 0.51; 2.68) p= 0.706 |

Legend: CRRT: Continuous renal replacement therapy, RRT: Renal replacement therapy

**Table S4: Clinical variables before discontinuation of continuous renal replacement therapy (CRRT) and before the first intermittent renal replacement therapy (IRRT) session in relationship with the occurrence of intra-dialytic hypotension.**

|  | **Variables** | **All transitions**  **(N=231)** | **No hypotension**  **(N=115)** | **Hypotension**  **(N=116)** | **p-value** |
| --- | --- | --- | --- | --- | --- |
| **Before CRRT discontinuation** | **Mechanical ventilation** | 205 (88.7%) | 100 (86.2%) | 105 (91.3%) | 0.220 |
|  | **Cumulative fluid balance (L)** | 7.4 (1.5; 14.1) | 5.4 (0.6; 12.6) | 9.5 (2.3; 15.9) | 0.036 |
|  | **24h urine output ( mL)** | 55 (5; 160) | 78 (25; 188) | 35 (0; 120) | 0.005 |
|  | **Time on CRRT (days)** | 5 (3; 10) | 5 (2; 9) | 6 (3; 13) | 0.009 |
|  | **Vasopressor use** | 95 (41.1%) | 33 (28.4%) | 62 (53.9%) | <0.001 |
|  | **Number of past transitions attempts** | 1 (1; 2) | 1 (1;2) | 2 (1; 2) | 0.001 |
| **Before IRRT initiation** | **Heart rate (beats/min)** | 91 ±19 | 89 ±18 | 92 ±20 | 0.238 |
|  | **sBP (mmHg)** | 118 (105; 137) | 130 (111; 146) | 110 (101; 127) | <0.001 |
|  | **dBP (mmHg)** | 58 (49; 66) | 61 (54; 71) | 55 (47; 63) | <0.001 |
|  | **Mechanical ventilation** | 201 (87.0%) | 94 (81.0%) | 107 (93.0%) | 0.007 |
|  | **Vasopressor use** | 102 (44.2%) | 33 (28.4%) | 69 (60%) | <0.001 |
|  | **Prescribed Relative fluid removal (% of BW)** | 2.1 (1.4; 3.0) | 2.1 (1.3; 3.1) | 2.2 (1.5; 2.9) | 0.763 |
|  | **Time gap between CRRT and IRRT (hours)** | 25 (15; 41) | 26 (16; 47) | 22 (15; 37) | 0.073 |
|  | **Prescribed treatment time (hours)** | 4 (4; 8) | 4 (4; 4.5) | 6 (4; 8) | <0.001 |

**Lengend:** BW: Body weight, 95%, CRRT: continuous renal replacement therapy, dBP: diastolic arterial blood pressure, IRRT: intermittent renal replacement therapy, sBP: systolic arterial blood pressure.

**Table S5: Association between elements of the IRRT prescription and IDH**

| **Prescription** | **OR (CI) p** |
| --- | --- |
| **Dialysate sodium**   - **136-140 mmol/L** - **≤ 135 mmol/L** - **>140 mmol/L** - **Sodium profiling** | Reference  0.86 (CI: 0.49; 1.51) p=0.60  0.98 (CI: 0.25; 3.87) p=0.98  0.48 (CI: 0.19; 1.27) p=0.14 |
| **Dialysate temperature**   - **36.0-37.0** - **<36.0** - **Temperature control** | Reference  2.04 (CI: 0.77; 5.38) p=0.15  0.95 (CI: 0.32; 2.78) p=0.93 |

Legend: Associations were assessed using Generalized estimating equations with a binary logistic link function and using an M-estimator with an independent correlation matrix.

**Table S5: Individual component of the SOFA score at the discontinuation of continuous renal replacement therapy (CRRT) and immediately before the initiation of intermittent renal replacement therapy (IRRT)**

| **Component of the SOFA score** | | **At CRRT interruption** | **Before IRRT initiation** |
| --- | --- | --- | --- |
| **Coagulation:**  **Platelet count**  **(×10^3^/μl)** | **0: ≥ 150** | 91 (39.4%) | 103 (44.6%) |
|  | **1: < 150** | 40 (17.3%) | 39 (16.9%) |
|  | **2: < 100** | 48 (20.8%) | 45 (19.5%) |
|  | **3: < 50** | 41 (17.7%) | 36 (15.6%) |
|  | **4: < 20** | 11 (4.8%) | 8 (3.5%) |
| **Liver function:**  **Total bilirubin**  **(μmol)** | **0: < 20** | 102 (44.2%) | 106 (45.9%) |
|  | **1: 20-32** | 29 (12.6%) | 29 (12.6%) |
|  | **2: 33-101** | 60 (26.0%) | 55 (23.8%) |
|  | **3: 33-101** | 21 (9.1%) | 18 (7.8%) |
|  | **4: > 204** | 19 (8.2%) | 23 (10.0%) |
| **Respiratory function: PaO2/FiO2 ratio** | **0: ≥ 400** | 33 (14.3%) | 22 (9.5%) |
|  | **1: < 400** | 61 (26.4%) | 56 (24.2%) |
|  | **2: < 300** | 76 (32.9%) | 85 (36.8%) |
|  | **3: < 200** | 55 (23.8%) | 55 (23.8%) |
|  | **4: < 100** | 6 (26.0%) | 13 (5.6%) |
| **Cardiovascular function: MAP and pharmacologic support** | **0: MAP ≥ 70 mmHg** | 108 (46.8%) | 99 (42.9%) |
|  | **1: MAP < 70 mmHg** | 28 (12.1%) | 34 (14.7%) |
|  | **2: Dopamine ≤ 5 μg/kg/min or dobutamine (any dose)** | 2 (0.9%) | 0 (0%) |
|  | **3: Dopamine > 5 μg/kg/min OR epinephrine ≤ 0.1 μg/kg/min OR norepinephrine ≤ 0.1 μg/kg/min** | 45 (19.5%) | 51 (22.1%) |
|  | **4: Dopamine > 15 μg/kg/min OR epinephrine > 0.1 μg/kg/min OR norepinephrine > 0.1 μg/kg/min** | 48 (20.8%) | 47 (20.3%) |
| **Neurologic function: Glasgow Coma Scale** | **0: 15** | 13 (5.6%) | 14 (6.1%) |
|  | **1: 13–14** | 10 (4.3%) | 11 (4.8%) |
|  | **2: 10–12** | 66 (28.6%) | 78 (33.8%) |
|  | **3: 6–9** | 61 (26.4%) | 51 (22.1%) |
|  | **4: < 6** | 81 (35.1%) | 77 (33.3%) |

Legend: CRRT: Continuous renal replacement therapy, IRRT: Intermittent renal replacement therapy, MAP: Mean arterial pressure

**Table S6: CRRT-to-IHD subgroup analysis: Variables associated with intra-dialytic hypotension (using Definition 2A)**

|  | **Variables** | **CRRT-to-IHD** | |
| --- | --- | --- | --- |
|  |  | **Univariable**  **OR (95%CI) p-value** | **Multivariable**  **Adj OR (95%CI) p-value** |
| **Model 1:**  **Before CRRT discontinuation** | **Mechanical ventilation** | 1.60 (0.58; 4.41) 0.367 | 1.29 (0.43; 3.87) 0.646 |
|  | **Cumulative fluid balance (per L)** | 1.03 (1.00; 1.06) 0.048 | 1.03 (1.00; 1.07) 0.051 |
|  | **24h urine output (per 100 mL)** | 0.92 (0.84; 1.02) 0.108 | 0.90 (0.81; 0.99) 0.039 |
|  | **Time on CRRT (per day)** | 1.03 (0.98-1.09) 0.290 | 1.01 (0.96; 1.06) 0.721 |
|  | **Vasopressor use** | 1.85 (0.88; 3.87) 0.104 | 1.96 (0.90; 4.26) 0.09 |
|  | **Number of past transitions attempts** | 1.79 (0.72; 4.44) 0.207 | 1.24 (0.41; 3.66) 0.700 |
| **Model 2:**  **Before IRRT initiation** | **Heart rate (per 10 beats/min)** | 0.97 (0.80-1.19) 0.802 | 1.10 (0.89; 1.37) 0.384 |
|  | **sBP (per 10 mmHg)** | 0.65 (0.49; 0.85) 0.001 | 0.77 (0.61; 0.98) 0.033 |
|  | **dBP (per 10 mmHg)** | 0.73 (0.48-0.84) 0.002 | 0.84 (0.60; 1.17) 0.299 |
|  | **Mechanical ventilation** | 3.24 (1.04; 10.1) 0.043 | 3.31 (1.05; 10.4) 0.033 |
|  | **Vasopressor use** | 2.98 (1.40; 6.32) 0.005 | 2.39 (0.93; 6.17) 0.072 |
|  | **Prescribed Relative fluid removal (% of BW)** | 1.10 (0.86-1.42) 0.450 | 1.27 (0.96;1.67) 0.093 |
|  | **Time gap between CRRT and IRRT (days)** | 0.99 (0.98-1.003) 0.128 | 0.99 (0.97; 1.01) 0.154 |
|  | **Prescribed treatment time (hours)** | 1.33 (0.76-2.31) 0.319 | 1.18 (0.66; 2.11) 0.579 |

Abbreviations: BW: Body weight, CI: Confidence interval, CRRT: Continuous renal replacement therapy, dBP: diastolic arterial blood pressure, IHD: Intermittent hemodialysis, sBP: systolic arterial blood pressure, OR: Odds ratio.

**Table S7: Severity of illness at the time of IRRT initiation according to IRRT modality.**

| Characteristics | CRRT-to-IDH | CRRT-to-SLED | p-value |
| --- | --- | --- | --- |
| Heart rate (beats/min) | 87 (73; 99) | 96 (85; 105) | 0.001 |
| sBP (mmHg) | 124 (108; 142) | 112 (101; 130) | 0.002 |
| dBP (mmHg) | 60 (50; 67) | 56 (48; 64) | 0.024 |
| Cumulative fluid balance (L) | 5.6 (1.0; 13.7) | 10.0 (4.3; 17.9) | 0.044 |
| Mechanical ventilation | 115 (82.7%) | 86 (93.5%) | 0.017 |
| SOFA score | 8 (7; 10) | 12 (9; 14) | <0.001 |
| Vasopressor support | 38 (27.3%) | 64 (69.6%) | <0.001 |
| VIS | 0 (0; 3.1) | 5 (0; 13) | <0.001 |

Values are in medians (IQR) or count (%). Abbreviations: SLED: slow low efficiency dialysis, dBP: diastolic arterial blood pressure, IHD: Intermittent hemodialysis, sBP: systolic arterial blood pressure, SOFA: sequential organ failure assessment, VIS: Vasoactive-inotropic score.

**Table S8: Revised IDH prediction model 2 including the receipt of SLED**

|  | **Variable** | **Univariable**  **OR (95%CI) p-value** | **Multivariable**  **Adj OR (95%CI) p-value** |
| --- | --- | --- | --- |
| **Model 2:**  **Before IRRT initiation** | **Heart rate (per 10 beats/min)** | 1.06 (0.92; 1.21) 0.432 | 1.09 (0.92; 1.30) 0.323 |
|  | **sBP (per 10 mmHg)** | 0.75 (0.65; 0.86) < 0.001 | 0.85 (-0.73; 1.00) 0.050 |
|  | **dBP (per 10 mmHg)** | 0.65 (0.51; 0.82) < 0.001 | 0.78 (0.59; 1.04) 0.095 |
|  | **Mechanical ventilation** | 2.93 (1.24; 6.89) 0.014 | 1.99 (0.77; 5.10) 0.151 |
|  | **Vasopressor use** | 3.95 (2.26; 6.91) < 0.001 | 2.36 (1.20; 4.68) 0.013 |
|  | **Prescribed Relative fluid removal (% of BW)** | 1.76 (0.25; 12.35) 0.571 | 1.31 (1.04; 1.63) 0.018 |
|  | **Time gap between CRRT and IRRT (days)** | 0.74 (0.57; 0.97) 0.029 | 078 (0.55; 1.10) 0.153 |
|  | **Use of SLED** | 4.10 (2.33; 7.22) <0.001 | 2.32 (1.18; 4.55) 0.015 |

Abbreviations: BW: Body weight, CI: Confidence interval, SLED: slow low efficiency dialysis, dBP: diastolic arterial blood pressure, IHD: Intermittent hemodialysis, sBP: systolic arterial blood pressure, OR: Odds ratio.
